# Neurophysiological basis for visual distortions in amblyopia

**DOI:** 10.3389/fnins.2026.1803396

**Published:** 2026-07-15

**Authors:** Laurie Goulet, Reza Farivar

**Affiliations:** 1 Department of Ophthalmology and Visual Sciences, McGill University, Montreal, QC, Canada; 2 Research Institute of McGill University Health Centre, Montreal, QC, Canada

**Keywords:** amblyopia, cortical processing, perceptual distortions, spatial integration, vision

## Abstract

Despite decades of research, the neural basis of visual distortions in amblyopia remains poorly understood. While numerous studies have reported abnormalities in both early visual cortex and extrastriate areas, these findings have rarely been integrated into a unified mechanistic framework. This review addresses that gap by proposing a dual-level model of cortical distortion in amblyopia: one level reflecting local feature encoding deficits in V1, and a second reflecting integration-level distortions in retinotopic extrastriate cortex. By synthesizing behavioral, neurophysiological, and imaging data from adult human studies as well as animal models, this review offers a coherent neurophysiological account of amblyopic visual distortions and outlines a framework for interpreting current findings and guiding future interventions.

## 1 Introduction

Amblyopia is a neurodevelopmental visual deficit that affects both children and adults and is one of the leading causes of visual loss in an otherwise healthy eye (Jakobsson et al., 2002; Donnelly et al., 2005; Robaei et al., 2006); Williams et al. (2008). It arises from a disruption of binocular vision during critical developmental periods in early life, leading to the brain favoring the eye with better vision (i.e., fellow fixing eye, FE) while neglecting the weaker one (i.e., amblyopic eye, AE) (Hensch and Quinlan, 2018). The most common causes of amblyopia are strabismus, anisometropia, or a combination of both. Anisometropic amblyopia results from unequal refractive errors between the two eyes, while strabismic amblyopia arises from misalignment of the eyes during development. Although amblyopia in both eyes can occur (Haase and Mühlig, 1979; Schoenleber and Crouch, 1987; Wallace et al., 2007; Birch, 2013), it is far less prevalent and less studied, and thus here we focus on monocular amblyopia.

**Figure 1:**
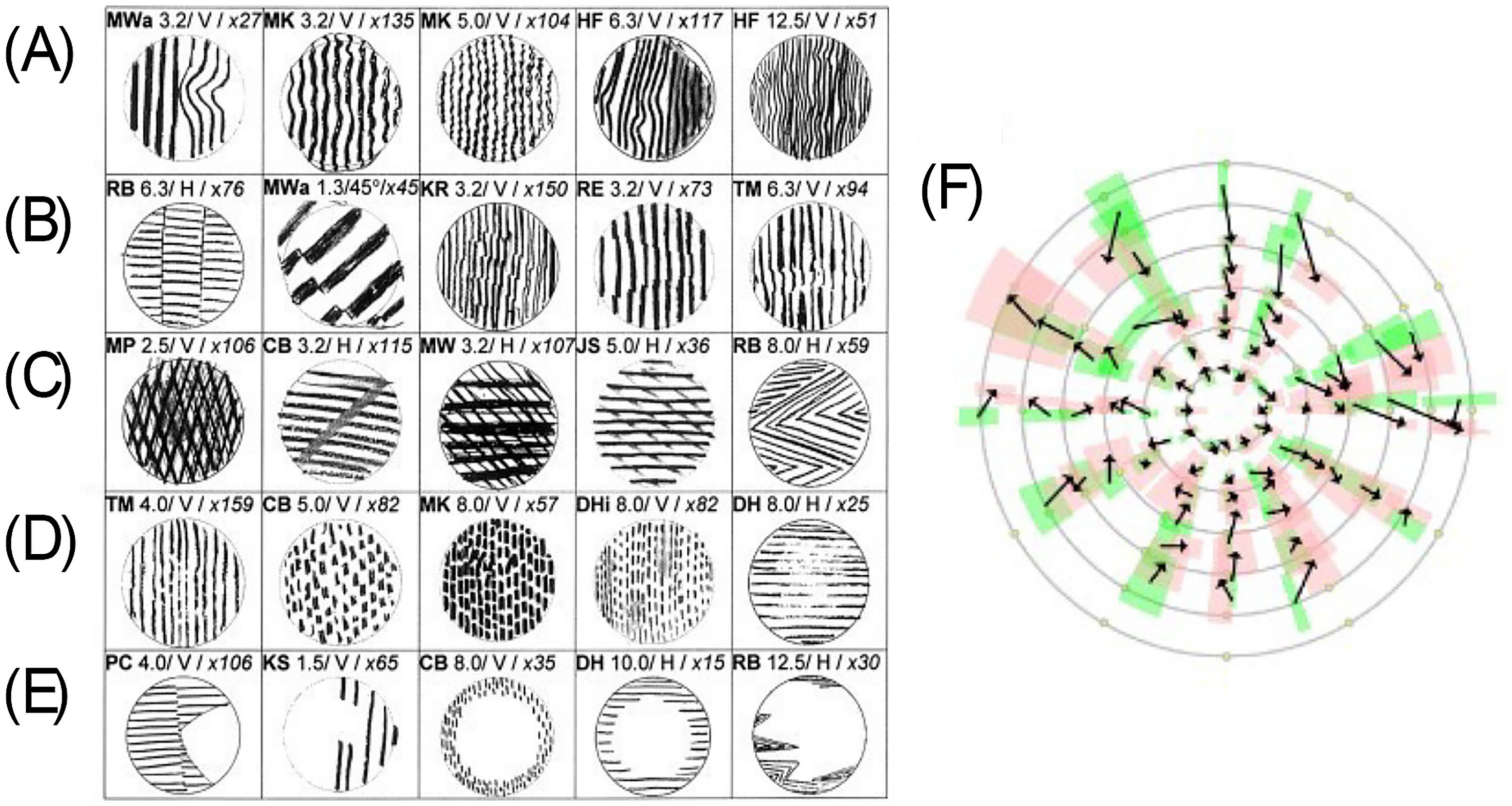
Examples of visual distortions perceived with the amblyopic eye. **(A–E)** Examples of the five main categories of visual distortions that emerge from sinusoidal gratings perception. **(A)** Examples of wavy distortions. **(B)** Examples of abrupt offset distortions. **(C)** Examples of misperceived orientation distortions. **(D)** Examples of fragmented distortions. **(E)** Examples of scotomatous distortions. **(F)** Example of displacement distortion using a circular pattern. Adapted from Barrett et al. (2003), Sireteanu et al. (2008b).

Symptoms of amblyopia can include impaired visual acuity, reduced stereoacuity, and contrast sensitivity deficits, especially at high spatial frequencies (SF), leading to challenges in recognizing fine details and patterns, as well as perceptual distortions (Hess et al., 1978; Barrett et al., 2003; Sireteanu et al., 2008b; Hess et al., 2014; Kwon et al., 2014a; Piano et al., 2015). Studies on patients’ perception of grating and dot pattern stimuli have revealed that around 67% of amblyopes report experiencing perceptual distortions in their AE (Barrett et al., 2003; Fronius et al., 2004; Piano et al., 2015, 2016). Patients describe their perception of gratings as jagged, fragmented, mis-oriented, scotomatous, or of wavy appearance (Figures 1A–E). When viewing dot pattern stimuli, distortions are reported as spatial displacement and spatial uncertainty (Figure 1F). Most of these studies found elevated levels of distortion levels in the AE, even among patients with otherwise mild clinical impairment.

Perceived visual distortions have been shown to be independent from other major visual disruptions in amblyopia and to persist despite improvements of visual acuity following “successful” treatment outcomes. For example, Piano et al. (2015) reported that some patients with close-to-normal visual acuity still experienced severe distortions in their AE. Relatedly, Piano et al. (2016) found no significant relationship between the severity of perceptual distortions and amblyopia treatment outcomes. More than half of the “treated” amblyopes still experienced substantial distortions in their AE despite post-treatment improvements in acuity, suggesting that distortions are at least partially independent from visual acuity losses and amblyopia severity. Consistent with this, Molaei et al. (2025b) found no significant correlation between distortion magnitude and visual acuity, reinforcing the idea that perceptual distortions likely originate from distinct mechanisms than visual acuity impairments.

It is important to note that the prevalence and nature of distortion come from estimates using laboratory made stimuli such as Gabor patches, sinusoidal gratings, dot pattern stimuli. While such highly-controlled stimuli have been instrumental to the bulk of vision research, they do not account for real-life experiences and there is increasing evidence that neural responses to such stimuli may be different than to natural scenes (Kayser et al., 2003; Felsen and Dan, 2005; Felsen et al., 2005; Touryan et al., 2005; Talebi and Baker, 2012). To this day, no research has been done to clearly estimate the nature and extent of spatial distortions using naturalistic stimuli. Attempts have been made at applying distortion patterns estimated from simple stimuli to more complex ones, but from reasons that make sense in light of how the visual system is organized and responds to various stimuli, none of them were conclusive at replicating exactly what amblyopes perceive (Sireteanu et al., 1993; Iftime et al., 2007).

Despite the prevalence of reported distortions, the neural mechanisms underlying these perceptual errors remain poorly understood. In particular, existing models do not yet provide a unified framework linking abnormalities in early visual cortex to the structured distortions experienced by amblyopes. Existing theories have primarily focused on early-stage encoding abnormalities, such as abnormal retinotopy, reduced contrast sensitivity, and degraded spatial resolution in primary visual cortex (V1). While these impairments are well documented, they do not fully account for the complexity, spatial diversity and stimulus dependence of the perceptual distortions described by amblyopic individuals. As a result, the cortical origin of these distortions remains an open question, one that cannot be fully addressed by considering V1 functional alterations alone.

Recent mechanistic accounts of amblyopic visual impairments can be broadly grouped into three classes: (1) compensatory pooling and gain-normalization models, (2) Bayesian decoding frameworks, and (3) abnormal retinotopic encoding. Compensatory pooling and gain-normalization models propose that distortions arise from changes in how neural responses are pooled or represented at the population-level. This first group of theories suggests that major rearrangements of cortical topography are not required to explain distortions. Olianezhad et al. (2025) proposed that weakened and unreliable AE inputs trigger a compensatory mechanism that increases the spatial extent over which neuronal responses are integrated in V1. This enlarged integration region is likely to recruit neurons with mismatched feature preferences (i.e., orientation, spatial phase, polarity). This mixing of local features could manifest perceptually as waviness, fragmentation, or contour deformation. In a related but quantitatively different framework, Zhu et al. (2025) explained amblyopic impairments by a right-shift of contrast response function (CRF) combined with a reduction in population Fisher information for orientation coding. In this model, population responses carry less stimulus information even when single-neuron tuning remains broadly intact, with information loss driven by contrast-dependent gain changes.

These models can explain threshold-level impairments by linking perceptual loss to contrast-dependent reductions in neural gain or population information. However, because both compensatory pooling and gain-normalization explanations are explicitly driven by weakened contrast responses, they predict that distortions should be significantly reduced at suprathreshold contrast. Contrary to this prediction, perceptual distortions in amblyopia are frequently observed at suprathreshold contrasts (Pugh, 1958; Sireteanu et al., 1993; Barrett et al., 2003; Molaei et al., 2025b), where interocular differences in V1 response amplitude are substantially reduced (Kiorpes et al., 1998; Acar et al., 2019a). Distortions can also occur in individuals with intact contrast sensitivity functions (Hess et al., 1978). In such cases, there is no longer a clear physiological trigger for compensatory pooling or contrast-dependent information loss. The persistence and structured nature of suprathreshold distortions are therefore difficult to reconcile with compensatory pooling or population-information losses alone.

A second group of hypotheses places the origin of distortion not in early visual encoding but in Bayesian or perceptual decoding mechanisms. Schluppeck et al. (2025) reported that retinotopic maps and cortical magnification in V1 are largely preserved in amblyopia and proposed instead that distortions originate from later stages of perceptual inference. In their model, signals from the AE are noisier, leading the visual system to rely more heavily on positional priors during perceptual decoding, which produces systematic spatial errors despite relatively preserved encoding. This class of models provides an important account of how degraded sensory signals may bias perceptual inference and contribute to global positional distortions. However, Bayesian decoding frameworks do not naturally explain the local waviness, contour fragmentation or dot-pattern distortions commonly reported by amblyopic observers. Positional priors are generally expected to stabilize perception, especially near the fovea. This makes it difficult for decoding-based models alone to account for the strong spatial heterogeneity and feature dependence of amblyopic distortions, which often extend well beyond the fovea (Lagrèze and Sireteanu, 1991; Sireteanu et al., 1993; Mansouri et al., 2009; Molaei et al., 2025b).

A third class of theories attributes amblyopic distortions to abnormal retinotopic encoding. Classical hypotheses of neural undersampling (Levi and Klein, 1983) and neural scrambling (Watt and Hess, 1987) propose that visual space in V1 is either represented with reduced sampling density of high-spatial-frequency AE neurons, or with disordered topographic wiring of AE neurons. More recent population receptive field (pRF) mapping studies extend this view by demonstrating systematically enlarged pRFs across V1**–**V3, particularly in foveal representations, which have been interpreted as evidence for abnormal cortical projections (Clavagnier et al., 2015; Szinte et al., 2024). Encoding-stage theories correctly emphasize that distortions must originate within retinotopically organized cortex. However, encoding-only models do not explain why relatively subtle pRF irregularities or wiring disruptions can give rise to large, stimulus-dependent, and spatially heterogenous distortions (Barrett et al., 2003; Sireteanu et al., 2008a). Several physiological studies demonstrate that V1 responses can remain substantially more intact than behavioral performance would predict (Movshon et al., 1987; Kiorpes et al., 1998; Shooner et al., 2015). This mismatch suggests that distortions cannot arise purely from degraded V1 encoding but must instead reflect downstream transformation and integration of early-stage irregularities within retinotopically organized extrastriate cortex. Together, these observations reveal a persistent gap between physiological measures of early visual cortex and the structured perceptual distortions reported by amblyopic observers. Existing models tend to focus on either early encoding abnormalities or higher-level perceptual interpretations, but rarely explain how distortions in early retinotopic maps are transformed through the visual hierarchy to produce the spatially structured percepts reported by patients.

In this review article, we argue that perceptual distortions reflect a dual-level cortical dysfunction. At the first level, early abnormalities in the lateral geniculate nucleus (LGN) and V1 degrade the fidelity of local features encoding for position, ocular dominance (OD), SF and, to a lesser extent, orientation. At the second level, distorted local signals from V1 are integrated within retinotopic extrastriate visual areas, where they are both amplified through spatial pooling and nonlinearly transformed by contextual and integrative processes into structured perceptual distortions.

## 2 The LGN is insufficient to explain distortions in amblyopia

### 2.1 The LGN likely contributes to distortions in amblyopia

Early work by Von Noorden et al. (1983); von Noorden and Crawford (1992) provided direct evidence of LGN cell shrinkage in human strabismic and anisometropic amblyopes using postmortem histology. The authors reported a decrease in cell sizes of the parvocellular layers innervated by the AE, the magnocellular layers showing no changes. Similar anatomical deficits have been reported in visually deprived and artificially induced strabismic monkeys (Headon and Powell, 1973; Von Noorden, 1973; Crawford and Von Noorden, 1979a, 1979b; Hendrickson et al., 1987; Sloper, 1993; Levitt et al., 2001).

In infant monkeys with surgically induced esotropia (i.e., monocular convergent strabismus), histological examination of the LGN revealed reduced neuronal sizes in laminae innervated by the AE. More specifically in monkeys with short-term esotropia, Crawford and von Noorden (1979b) reported a shrinkage of 6–8% in cells of the parvocellular layers connected with the AE compared to the FE, with no significant changes in the magnocellular layers. A more drastic difference in cell size (i.e., 20%) was present in one monkey with long-term esotropia. However, in monkeys these morphological deficits do not correlate very well with neurophysiological measures of LGN activity. Through extracellular microelectrode recordings of LGN parvocellular neuron, Sasaki et al. (1998) found that despite reported structural changes in cell size, units responses to drifting sinusoidal gratings were normal regardless of SF, temporal frequency or contrast. In monocularly deprived monkeys, general activation patterns (i.e., visual latency, patterns of spike discharge) and spatial properties (i.e., spatial resolution, size of receptive field center) of LGN cells innervated by the AE in reaction to stationary, square-wave modulated, phase-reversing sinusoidal gratings and drifting gratings, respectively, were unaffected by deprivation (Blakemore and Vital-Durand, 1986). It’s possible that these authors did not observe differences because of their choice of stimuli, as naturalistic stimuli (in contrast to synthetic stimuli) have been shown to affect visual neural responses differently (Kayser et al., 2003; Felsen and Dan, 2005; Felsen et al., 2005; Touryan et al., 2005; Talebi and Baker, 2012).

A similar but slightly different picture emerges from studies involving kittens with lid suture or surgically induced strabismus. Guillery (1972, 1973) reported a small reduction in growth of LGN neurons in visually deprived cats, specifically at the boundaries between regions receiving inputs from AE and FE. In cats with surgically produced squint, Tremain and Ikeda (1982) used flashing spots of light to assess receptive field position and properties of LGN and areas 17 (i.e., striate cortex) cells. Single-cell recording revealed that cell shrinkage in LGN layers receiving input from the AE also correlated with OD shifts in the visual cortex. Thus, greater LGN cell shrinkage was associated with increased dominance of the FE in the cortex.

In contrast to studies involving amblyopic monkeys, kittens also showed functional alterations in LGN properties. Ikeda and Wright (1976) found poorer spatial resolution (i.e., peak frequency activation to a sinusoidal grating) in some AE-driven LGN neurons representing central retina of kittens with artificial convergent squint. Reduced resolving power was equivalent to that of cells representing peripheral retina (7–8^o^eccentricity) of the FE. These results are directly related to the reduced foveal visual acuity which is one of the primary symptoms of amblyopia. They also compared the temporal properties of LGN cells, measured as response latency to an optimized stimulus located at the receptive field center. AE-dominated LGN cells responded with a delayed activity onset.

These latter findings align with the well-established temporal processing transformations that occur along the retina-LGN-V1 pathway (Hawken et al., 1996; Sincich et al., 2009; Uglesich et al., 2009; Wang et al., 2010; Alexander et al., 2022). In normal vision, the LGN transforms retinal inputs into more temporally precise signals through multiple mechanisms. First, temporal LGN responses are more transient than their retinal inputs. The LGN filters out very slow temporal fluctuations, effectively shifting from low-pass to band-pass temporal tuning (Stevens and Gerstein, 1976; Cleland and Lee, 1985; Usrey et al., 1999). In addition, the LGN exhibits both lagged and non-lagged response modes (Mastronarde, 1987; Humphrey and Weller, 1988; Saul, 2008). These modes are generated through feedforward inhibition mediated by LGN interneurons (Vigeland et al., 2013) and introduce systematic temporal offsets that decorrelate neural activity (Dong and Atick, 1995). These temporal transformations in the LGN have been suggested as possible mechanisms to improve timing precision and the reliability of motion-related signals sent to the cortex (Saul and Humphrey, 1990). When AE-driven LGN neurons show delayed latencies and reduced transience, as observed in kittens, the timing of the feedforward input becomes less reliable. This temporal imprecision aligns with known temporal behavioral abnormalities in amblyopia, including reduced temporal resolution (Spang and Fahle, 2009; Hu et al., 2021), impaired motion sensitivity (Kiorpes et al., 2006), and decreased synchrony (Huang et al., 2012; Tao et al., 2019). Changes in the timing and transience of LGN outputs could contribute to downstream defective temporal frequency processing in amblyopia.

Building on histological results in animal models, functional changes in humans also appear to be layer-specific. Early functional magnetic resonance imaging (fMRI) studies involving adults with anisometropic amblyopia reported reduced LGN activation in response to AE stimulation when compared to the FE and normally-sighted individuals (Miki et al., 2003; Hess et al., 2008; Hess et al., 2009b). High-resolution fMRI combined with pathway selective stimuli (i.e., a spatiotemporal narrowband radial gratings differing in chromaticity) in humans strabismic (*N* = 3) and anisometropic (*N* = 4) amblyopia later revealed that these changes were specific to the parvocellular layers of the LGN, which are important for processing fine details and forms (Hess et al., 2010b). Subsequent research revealed that despite AE-driven parvocellular functional reductions, manually defined anatomical LGN volumes did not differ between amblyopes and controls (Wen et al., 2021). Such parvocellular-specific reductions in activation could partially explain some of the visual impairments experienced by amblyopes, especially in tasks requiring fine spatial discrimination.

Contrast gain control in the LGN provides a potential subcortical contribution to contrast sensitivity losses in amblyopia (Freeman and Thibos, 1975; Bradley and Freeman, 1981; Wei et al., 2024). In normal vision, LGN neurons rescale their responses to visual contrast through strong contrast-dependent gain control (Carandini and Heeger, 2012) and extraclassical surround suppression (Jeffries et al., 2014). This way, each neuron’s output is adjusted relative to the overall level of activity in the surrounding neural population to maintain sensitivity across a wide range of contrasts. Although this intrinsic contrast sensitivity is strongest in magnocellular layer neurons in monkeys (Alitto and Usrey, 2008; Camp et al., 2009) and in Y cells in cats (Derrington and Felisberti, 1998), high-contrast achromatic stimuli in humans evoke strong BOLD response in the parvocellular layers of the LGN (Qian et al., 2020). This parvocellular response to high contrast stimuli is selectively reduced for the AE (Wen et al., 2021). Thus, even if magnocellular contrast sensitivity is largely preserved, weakened parvocellular gain at high contrast may reduce the effective contrast of signals delivered to the cortex and contribute to the behavioral contrast sensitivity losses observed in amblyopia.

### 2.2 The LGN alone cannot explain amblyopic visual distortions

As previously seen, the structural and functional alterations observed in the LGN in amblyopia suggest it plays a more complex role in early visual development than previously thought. Recent work shows that the LGN is more than a smart-gating relay center (Hubel and Wiesel, 1961): it can also act as a nonlinear filter of visual signals (Alitto and Usrey, 2003). In the LGN, feedback synapses outnumber retinal ones, with only around 5–10% of cells in the LGN receiving feedforward transmission from the retina (Guillery, 1971; Sherman and Guillery, 2002; Wilson, 2008). The rest are modulatory and are mediated by local inhibitory inputs, feedback inputs from layer 6 of V1, and ascending inputs from the brainstem (Bloomfield and Sherman, 1988; Wilson et al., 1995; Callaway, 1998; Sherman and Guillery, 2002; Hirsch and Martinez, 2006; Hirsch et al., 2015). It is important to note that this “modulator role” of the LGN is greatly reduced in the presence of anesthesia (Wrobel et al., 1984; Alitto et al., 2011). This could partially explain the lack of correlation between histological abnormalities and functional properties of LGN neurons in amblyopic animals previously reported, as much of the animal neurophysiology was conducted under anesthesia.

While LGN neurons are monocular, their responses are modulated by binocular cortical feedback. This provides a pathway through which abnormal interactions in amblyopia may influence thalamic response. At the same time, LGN feature representations—SF, orientation bias, motion-related timing—are present but weak (Derrington and Lennie, 1984; Hu et al., 2000; Xu et al., 2002; Tan and Yao, 2009; Viswanathan et al., 2011; Vidyasagar et al., 2015), and dominated by cortical feedback rather than intrinsic computations. Thus, while LGN abnormalities likely degrade the fidelity of feedforward signal, they cannot by themselves generate the pronounced losses in spatial resolution, contrast sensitivity and orientation discrimination seen behaviorally (Levi and Klein, 1982a, 1982b; McKee et al., 2003).

Taken together, the evidence suggests that LGN abnormalities in amblyopia reflect a combination of reduced parvocellular input, altered neural response amplitude and temporal filtering, and cortically driven modulations, but that the dominant sources of perceptual distortions arise in the cortex, where binocular integration, feature tuning and spatial interactions are expressed.

## 3 V1 is necessary but not sufficient to explain distortions in amblyopia

### 3.1 V1 feature maps: structure and integration

**Figure 2:**
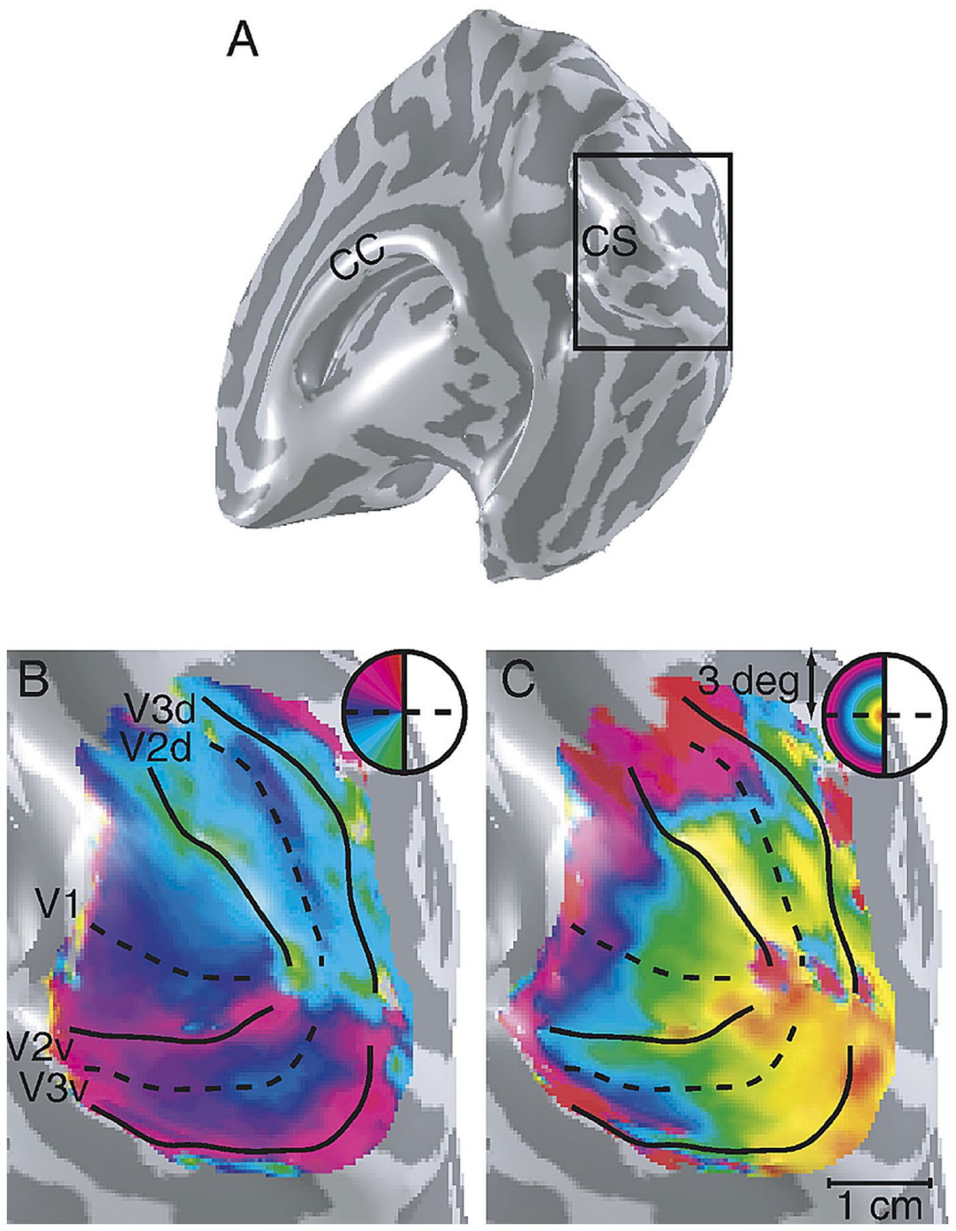
Retinotopic map in human V1. Retinotopic map showing the orderly representation of visual field location across the calcarine cortex **(A)**, with polar angle **(B)** and eccentricity **(C)** displayed on an inflated cortical surface. Adapted from Dumoulin and Wandell (2008).

**Figure 3:**
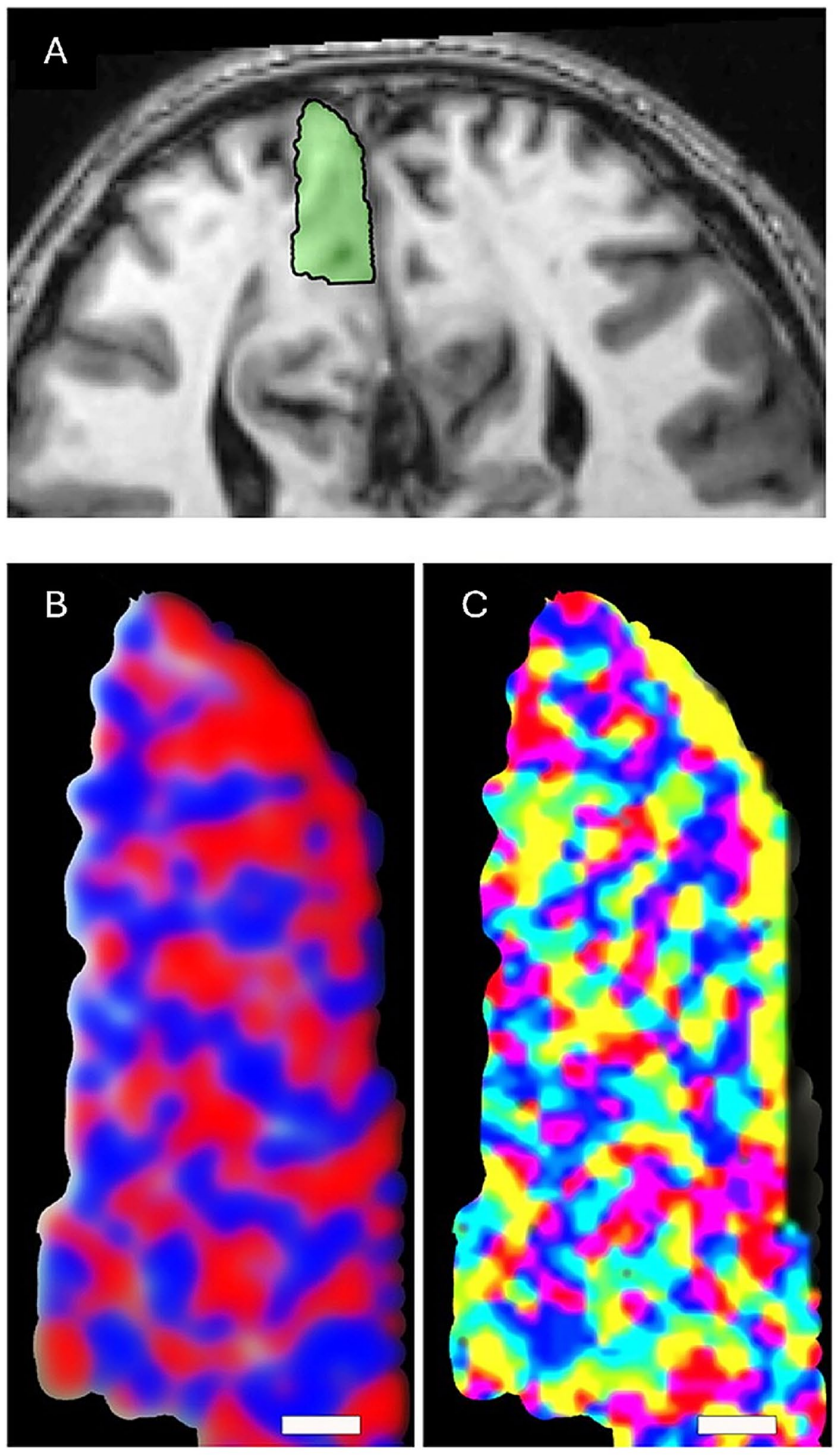
Ocular dominance and orientation maps in human V1. Ocular dominance **(B)** and orientation **(C)** maps of the ROI **(A)** representing a subsection of V1. Reproduced from Yacoub et al. (2008), Copyright (2008) National Academy of Sciences.

**Figure 4:**
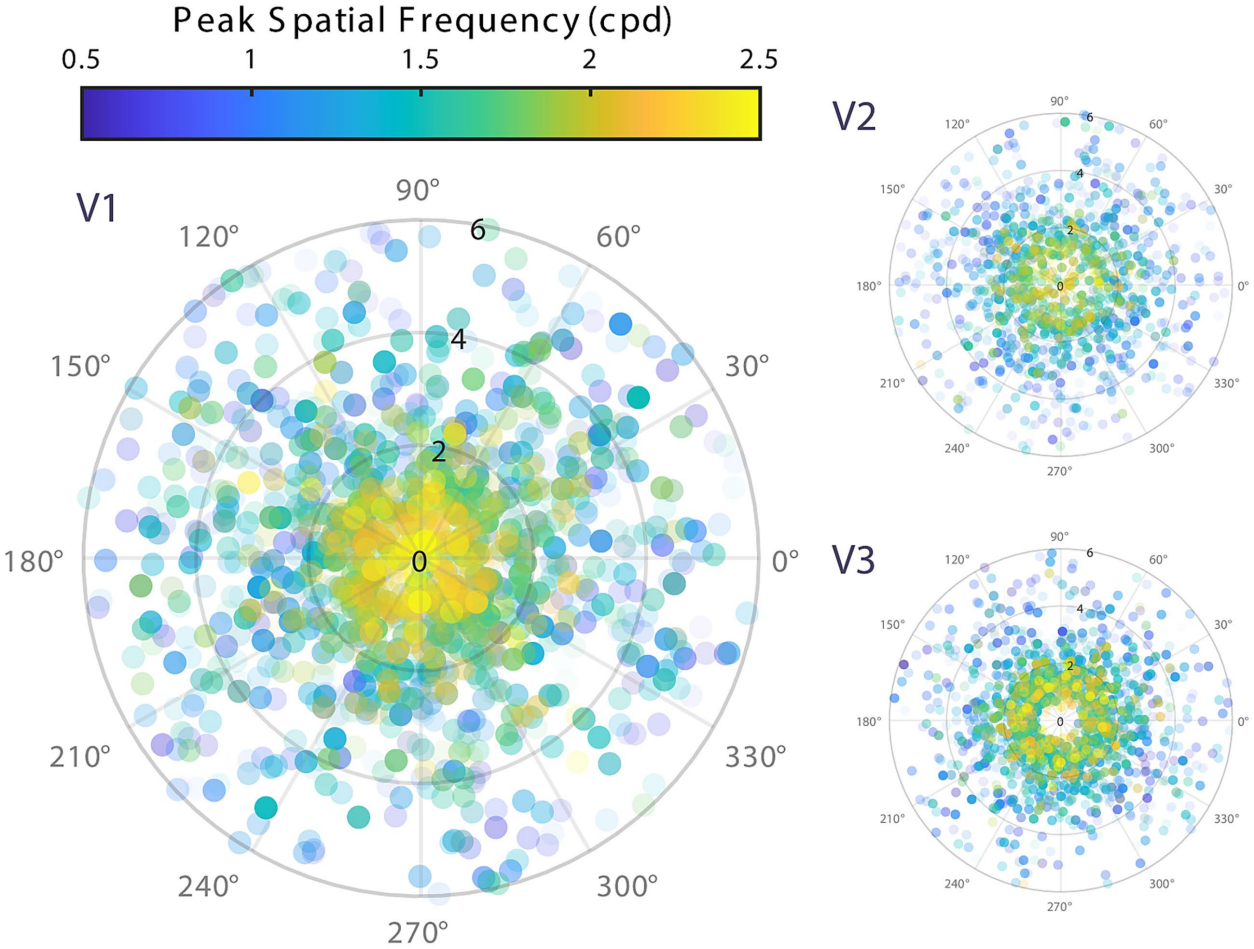
Spatial frequency representation in human V1. Peak spatial frequency across the visual field representation of early visual cortex (V1-V3). Reproduced from Goulet and Farivar (2025), Journal of Neurophysiology, licensed under CC-BY-NC-ND 4.0.

V1 maintains a retinotopic organization, where neighboring neurons correspond to neighboring regions of the visual field (Figure 2; Hubel and Wiesel, 1962; Dumoulin and Wandell, 2008). Within this retinotopic framework, neurons are further organized into OD columns representing inputs from each eye (Figure 3B; LeVay et al., 1985; Yacoub et al., 2007), orientation columns selective for bars or edges of specific angles (Figure 3C; Hubel and Wiesel, 1968; Yacoub et al., 2008), and spatial-frequency channels tuned to periodic changes in luminance (Figure 4; Movshon et al., 1978; Broderick et al., 2022). These maps do not exist in isolation, rather they overlap and interact. They form an orderly representation of the visual world in which each small cortical region contains populations of neurons spanning multiple visual feature preferences. For example, each retinotopic location contains a full set of orientation and OD columns (Hubel and Wiesel, 1977; Blasdel and Salama, 1986; Blasdel et al., 1995; Kim et al., 1999).

This architecture provides the fine-grained spatial precision required for normal vision but also introduces potential vulnerabilities. Even subtle misalignment in retinotopy, broader orientation tuning or reduction in SF selectivity could disrupt the fidelity of local visual feature encoding, leading to perceptual distortion downstream. The following section examines evidence from animal studies detailing how these low-level visual feature deficits emerge in amblyopia.

### 3.2 Local encoding of V1 neurons is affected in animal models of amblyopia

Disrupted visual input during the critical period of development has been shown to modify the functional architecture of V1 feature maps. Pioneering work by Wiesel and Hubel (1963, 1965), revealed that visual deprivation in kittens, achieved through eyelid suture, led to a loss of binocular responsiveness and reduced activity in V1 neurons driven by the deprived eye. Ocular dominance columns, normally equally distributed between the two eyes, shifted toward the non-deprived eye. These early findings indicated that patterned visual input during the critical period is essential for maintaining normal binocular function.

Although less extreme than full deprivation, both anisometropic and strabismic amblyopia disrupt V1 functional organization, particularly within V1 layers receiving parvocellular-dominated inputs that are critical for fine spatial processing. In animal models, these forms of amblyopia lead to measurable disruptions across several V1 feature maps, including retinotopic organization, OD, orientation selectivity, and SF tuning.

#### 3.2.1 Retinotopic disorganization in amblyopic animals

Both anisometropic and strabismic amblyopia result in abnormal retinotopy, particularly in central visual field representation. Swindale and Mitchell (1994) recorded from V1 in bilaterally amblyopic cats and found that receptive fields were, on average, 24% larger when compared to control animals. Similarly, in kittens with surgically induced unilateral strabismic amblyopia, AE-driven receptive fields were significantly larger (i.e., lower resolving power), and some binocular neurons presented enlarged, diffuse receptive fields with poorly defined boundaries (Baker et al., 1974; Yinon, 1975, 1976; Berman and Murphy, 1981; Chino et al., 1983). In macaques with experimentally induced anisometropia and strabismus, Kiorpes et al. (1998) observed broader receptive fields, especially in foveal areas corresponding to the AE. These findings suggest that retinotopic mapping is less precise in amblyopia. Given the interdependence between V1 feature maps, alterations in V1 retinotopy are likely to reflect broader disruptions in cortical organization (Blasdel and Campbell, 2001; Yu et al., 2005).

#### 3.2.2 Ocular dominance shifts in amblyopic animals

**Figure 5:**
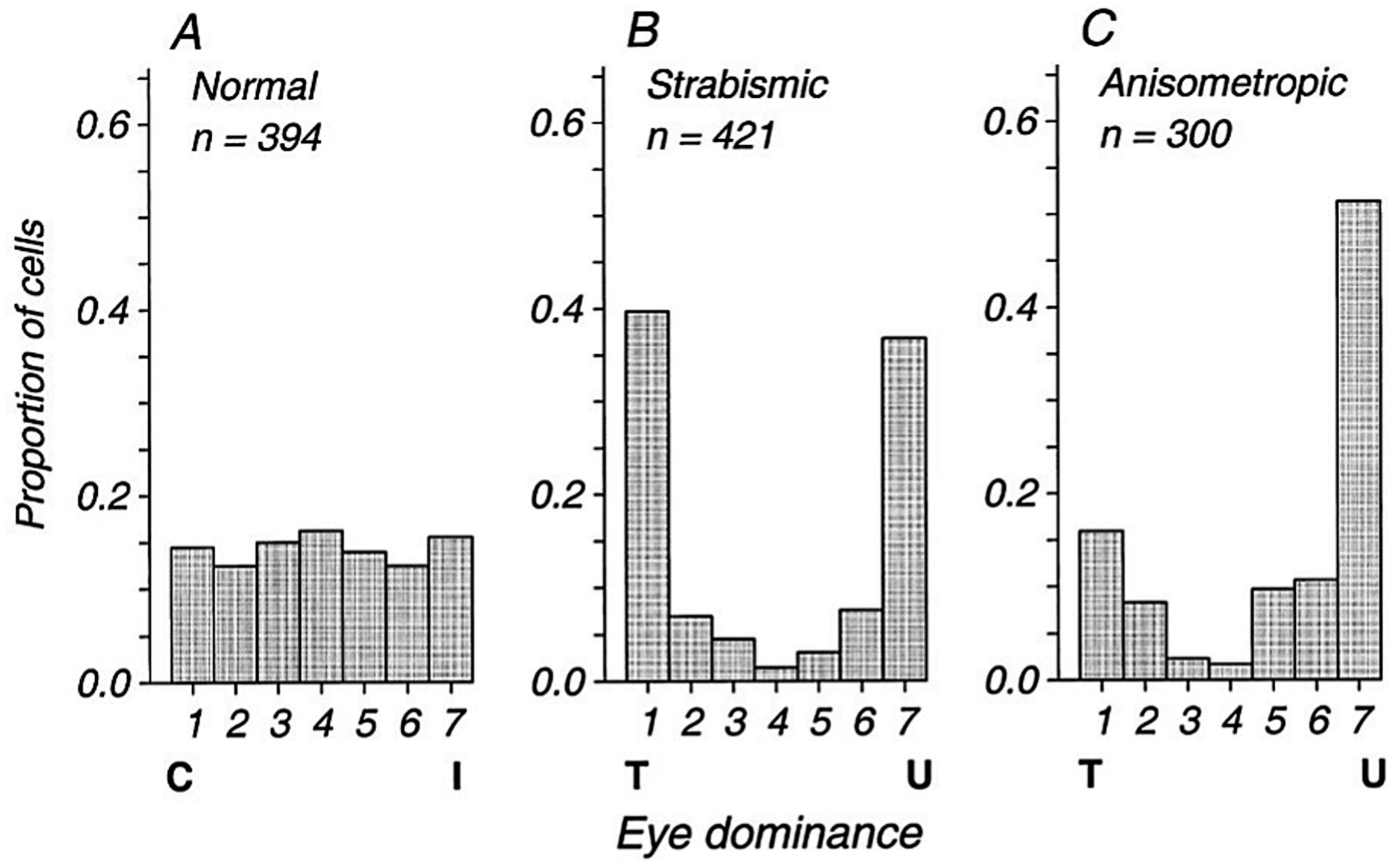
Cortical eye-dominance distribution in normal and amblyopic monkeys. **(A)** Normal control group showing balanced distribution of OD, where group 1 represents dominance by the contralateral eye (C) and group 7 by the ipsilateral eye (I). **(B)** Strabismic amblyopes exhibit a pronounced shift toward the treated eye (T) or untreated eye (U), with few binocularly balanced neurons. **(C)** Anisometropic amblyopes show a similar but asymmetric pattern favoring the untreated eye (U). Only neurons with receptive fields representing the central visual field were included. Adapted from Kiorpes et al. (1998), Copyright (1998) Society for Neuroscience.

In animal models, amblyopia systematically alters OD maps of V1. In a single macaque monkey with naturally occurring anisometropic amblyopia, Horton et al. (1997) reported reduced activity along the column borders, regions normally associated with binocular vision. In macaque monkeys with experimental anisometropia, Kiorpes et al. (1998) similarly documented a shift in OD toward the FE, marked by reduced AE responsiveness and widespread binocularity loss (Figures 5A,C). These results have been consistently reported across studies involving both monkeys (Hendrickson et al., 1987; Movshon et al., 1987; Horton and Stryker, 1993; Smith et al., 1997; Crawford and Harwerth, 2004; Shooner et al., 2015), and cats (Eggers and Blakemore, 1978; Maguire et al., 1982).

Similarly in non-human primates and cats, strabismic amblyopia produces a distinctive U-shaped OD distribution, reflecting a sharp reduction in binocular neurons (Figures 5A,B; Hubel and Wiesel, 1965; Baker et al., 1974; Blakemore and Eggers, 1978; Crawford and von Noorden, 1979a; Tremain and Ikeda, 1982; Freeman and Tsumoto, 1983; Kalil et al., 1984; Chino et al., 1991; Smith et al., 1997; Kiorpes et al., 1998; Schröder et al., 2002). Although AE-driven neurons are also reduced in strabismic amblyopia, the overall OD shift tends to be somewhat less extreme than in anisometropia, though substantial AE suppression is still commonly observed (Berman and Murphy, 1981; Cynader et al., 1984; Von Grünau, 1984; Acar et al., 2019b). Thus, strabismus seems to primarily affect binocular integration, while anisometropia additionally reduces AE-driven neurons’ activity.

#### 3.2.3 Abnormal spatial frequency tuning in amblyopic animals

**Figure 6:**
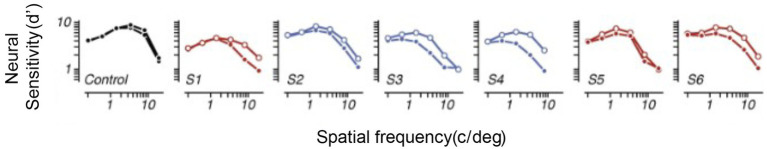
Spatial frequency tuning from single-cell recordings in amblyopic monkeys. Average neural sensitivity is plotted as a function of spatial frequency for control animals (black), strabismic (blue) and anisometropic (red) monkeys. Each column represents data from a different animal, comparing responses from the AE (filled symbols) and the FE (open symbols). AE responses showed reduced sensitivity, especially at high SF. Adapted from Shooner et al. (2015).

SF tuning is often impaired in amblyopic animals, with AE-driven neurons showing reduced sensitivity to high SFs and broader tuning. In infant monkeys with optically induced anisometropia, multiple studies reported reduced optimal SF and a selective loss of neurons tuned to high SF (Figure 6; Movshon et al., 1987; Kiorpes et al., 1998; Shooner et al., 2015). Similarly, AE neurons in kittens exhibited lower SF cutoffs, approximately one octave below those measured for the FE (Eggers and Blakemore, 1978).

Strabismic amblyopia also disrupts SF selectivity. Shooner et al. (2015) reported that in macaques, AE neurons presented reduced optimal SFs and diminished resolution (Figure 6). Optical imaging and Electrophysiological studies in cats confirmed weaker cortical activation for mid to high SF stimuli, with AE neurons showing reduced cutoffs and broader tuning (Chino et al., 1983; Crewther and Crewther, 1990; Chino et al., 1991; Schmidt et al., 2004). Notably, in some models, these abnormalities also extended to neurons driven by the fellow eye, challenging the notion that the non-amblyopic eye remains unaffected in amblyopia.

#### 3.2.4 Orientation map impairments in amblyopic animals

Orientation selectivity impairments in amblyopia remain a topic of debate, with animal studies reporting mixed findings. Electrophysiological work in amblyopic kittens and monkeys reported largely preserved orientation tuning in AE-driven neurons (Berman and Murphy, 1981; Movshon et al., 1987; Swindale and Mitchell, 1994; Kiorpes et al., 1998). Consistent with these findings, optical imaging studies in strabismic cats have shown that global organization of orientation maps remains largely intact (Löwel et al., 1998). However, subsequent research revealed subtle impairments, particularly in strabismic animals. These include reduced sharpness in orientation tuning (Chino et al., 1983) and reduced orientation selectivity in binocular neurons (Yinon, 1975, 1976).

Several studies have also pointed to a consistent pattern of reduced proportion of vertically tuned V1 neurons in strabismic amblyopic kittens, particularly in animals with marked loss of binocular units (Singer et al., 1979a; Singer et al., 1979b; Chino et al., 1991). Researchers attributed these impairments to impaired binocular fusion and vergence control. Cynader et al. (1984) also noted fewer vertically tuned V1 neurons in strabismic kittens regardless of the eye driving them, suggesting a cortical-level disruption rather than an input-driven one.

#### 3.2.5 Overview of animal models

Animal models of amblyopia demonstrate that early abnormal visual experiences lead to widespread alterations in V1 functional organization. Across retinotopy, OD, SF and orientation maps, AE-driven neurons consistently show impaired tuning and organization. Although the degree of impairment varies across amblyopia subtype and severity, findings from animal data highlight the potential link between V1 cortical map fidelity and perceptual abnormalities in amblyopia. The disruptions seen in animal models establish a foundation for understanding human amblyopia.

### 3.3 Human data is similar to animal models

Research using animal models has provided extensive evidence of altered cortical organization and tuning in amblyopia – such as disrupted retinotopy, abnormal OD, degraded orientation and SF tuning. Advances in functional MRI (fMRI), electroencephalography (EEG), and magnetoencephalography (MEG) have enabled researchers to investigate the organization and responsiveness of the human amblyopic visual cortex with growing precision. These studies reveal striking similarities with animal findings, suggesting that amblyopia is associated with both impaired visual selectivity and disordered neuronal organization in V1.

As in animal models, these functional impairments reflect not only altered spatial organization of feature maps but also reduced response reliability in AE-driven cortex. Several visual evoked potential (VEP) and neuroimaging studies report increased signal variability, lower signal-to-noise ratio (SNR), as well as attenuated and delayed response dynamics in adults with amblyopia (Anderson et al., 1999; Farivar et al., 2011; Kelly et al., 2015; Lygo-Frett et al., 2022; Wang et al., 2025). This suggests that heightened neural noise could contribute to degraded visual encoding in human amblyopia, but there is much variability to the definition of “neural noise” (Faisal et al., 2008).

#### 3.3.1 Disrupted retinotopic map organization in human amblyopia

Early imaging studies in amblyopic patients using positron emission tomography (PET) and single photon emission computed tomography (SPECT) reported reduced V1 activation to AE stimulation compared to the FE (Demer et al., 1988; Kabasakal et al., 1995). More recently, high-field fMRI has enabled finer-grained analyses of V1 retinotopy, showing impaired receptive field structure, and increased interocular deviation in the peak positions of stimulus activation (Dumoulin and Wandell, 2008; Chang et al., 2025).

Population receptive field (pRF; Dumoulin and Wandell, 2008) mapping revealed that AE-driven voxels exhibit larger pRF sizes across V1**–**V3, despite normal cortical magnification (Li et al., 2007b; Clavagnier et al., 2015; Lygo, 2019). These effects are seen across amblyopia subtypes (i.e., strabismic, anisometropic, and mixed) and are especially pronounced in foveal stimulation (Szinte et al., 2024). Clavagnier et al. (2015) attributed these results to disordered position of AE pRF. Li et al. (2007b) performed a phase-encoded retinotopic mapping and reported greater phase variance in AE-driven responses, results that they also interpreted as markers of spatial disorganization.

To determine whether spatial distortions in amblyopia were rooted in reduced neural responsivity or disrupted cortical organization, Farivar et al. (2019) quantified the spatial displacement between AE and FE responses in V1. Specifically, using fMRI they evaluated two metrics: (1) the interocular ratio of %BOLD signal change, and (2) the interocular deviation, defined as the distance between the cortical locations of peak activation for each eye. Amblyopic participants showed significantly reduced BOLD signal magnitudes for the AE across eccentricities and early visual areas. Additionally, interocular deviation scores were larger in amblyopes than for controls and correlated with amblyopia severity, suggesting increased scatter in the spatial representation of the AE. These deviations were present across a wide range of eccentricities, reflecting a global disruption of retinotopy in amblyopia rather than a fovea-specific issue.

#### 3.3.2 Ocular dominance imbalance in human amblyopia

Consistent with animal models, amblyopia leads to a shift in OD within human V1. Psychophysical studies have long suggested that binocular interactions are abnormal in amblyopia. Dichoptic contrast-balancing and binocular phase-combination paradigms have shown that signals from the AE contribute less strongly to binocular perception and are suppressed by the FE (Huang et al., 2009; Li J. et al., 2011; Ding et al., 2013; Zhou et al., 2013; Webber et al., 2020). Similarly, binocular rivalry experiments reveal prolonged suppression of AE input and reduced binocular integration (Handa et al., 2004; Chow et al., 2021; Hu et al., 2024). Together, these findings suggest that ocular dominance imbalance in amblyopia is expressed behaviorally through altered binocular combination and interocular suppression.

Neuroimaging findings are broadly consistent with these psychophysical observations. Using fMRI, several groups have reported reduced number of voxels responding to AE stimulation, along with lower BOLD signal amplitudes and a smaller cortical area activated by the AE (Goodyear et al., 2000; Algaze et al., 2002; Goodyear et al., 2002; Liu et al., 2004; Li et al., 2007a). Using high-resolution (7 T) imaging, Nasr et al. (2025) examined the organization of OD and found a consistent increase in the size of FE-driven cortical region in V1 across all amblyopia subtypes (i.e., strabismic, anisometropic, deprivation amblyopia). The effect was more pronounced in anisometropic subjects, especially in the hemisphere contralateral to the FE. This hemispheric asymmetry is consistent with the known imbalance in retinal ganglion cell density between nasal and temporal retina (Perry et al., 1984; Perry and Cowey, 1985), which results in stronger feedforward input from the FE to the contralateral hemisphere. In amblyopia, this pre-existing asymmetry may bias competitive cortical reorganization toward an expanded FE representation in contralateral V1. A reduction in binocularly driven voxels within V1 was also documented in amblyopia, with strabismic individuals exhibiting a lower proportion of binocular activation (16.85 ± 2.98%) than anisometropic amblyopes (34.98 ± 4.60%, ρ <0.05; Lee et al., 2001; Conner et al., 2007). reported that AE stimulation results in both delayed and reduced BOLD response in V1 for the AE stimulation, particularly when the FE was viewing a static pattern. Their results suggest that amblyopia not only reduces cortical responsiveness to AE stimulation but also enhances interocular suppression. Beyond OD shifts, human studies also demonstrate weak binocular facilitation and strong interocular suppression in V1 (Farivar et al., 2011; Lygo et al., 2021; Wang et al., 2024), suggesting that binocular interaction imbalances could substantially contribute to the cortical imbalance in amblyopia.

#### 3.3.3 Impairments of spatial frequency tuning in human amblyopia

Reports of SF tuning impairments in human amblyopia have been mixed, with results varying across amblyopia subtypes and methodology employed. Early fMRI studies, such as Goodyear et al. (2000) found no consistent relationship between SF and cortical activation in the calcarine cortex. However, more recent work has revealed amblyopia subtype-specific alterations in SF processing. Choi et al. (2001) showed reduced AE-driven activation to mid-high SF (0.5–2 cycles per degree of visual angle, cpd) in anisometropic amblyopia, and to low SF (0.25–1 cpd) in strabismic amblyopia. Similarly, Lee et al. (2001) found overall lower voxel activation in strabismic compared to anisometropic amblyopes, with selective attenuation of high SF (0.5–2 cpd) responses in the AE of the anisometropic group.

Other studies have examined SF tuning at a finer scale. Hess et al. (2009a) reported that in strabismic and mixed amblyopes, SF maps driven by the AE were less orderly and more spatially fragmented than those driven by the FE or controls. This “patchiness” reflected a reduction in the number of voxels exhibiting reliable SF preference resulting in noisier SF maps. Extending this line of work, Wiecek et al. (2023, 2024) reported reduced selectivity to high SF and broader tuning curves for AE responses, particularly within the central 4^o^ of visual field. Using MEG, Anderson et al. (1999) found that AE-driven responses to chromatic (red/green) sinusoidal gratings were delayed and reduced in amplitude across the SF spectrum. Even though the overall shape of the SF tuning curve was preserved, the overall magnetic field power was consistently reduced for the AE. These results suggest amblyopia disrupts SF processing at both threshold and suprathreshold levels where visibility is no longer limiting sensitivity.

#### 3.3.4 Overview of human data

Findings from human studies align closely with animal models. Disrupted retinotopic organization, OD imbalance, and degraded SF tuning all point to widespread alterations in early visual cortical architecture and function. These converging results reinforce the view that amblyopia is a cortical disorder rooted in disrupted feature map encoding and reduced response precision.

### 3.4 V1 alone cannot explain distortions in amblyopia

Animal neurophysiology and neuroimaging jointly suggest that V1 neurons driven by the AE present reduced sensitivity and abnormal feature tuning. Although V1 alterations are well documented, they tend to be relatively modest and are often too small to fully account for the magnitude and structure of perceptual distortions that we see in amblyopes.

Several studies have compared the magnitude of physiological changes in V1 with their behavioral equivalent. Movshon et al. (1987); Kiorpes et al. (1998) reported that although V1 neurons in monkeys with experimentally induced strabismus or anisometropia show reduced contrast sensitivity and spatial resolution, these changes do not map cleanly onto behavioral measures. For example, in some severely amblyopic animals, V1 neurons continued to respond to SFs that were perceptually invisible to the animal as assessed behaviorally. Although interocular ratios of spatial resolution and optimal frequency of V1 neurons correlated with the severity of amblyopia, as captured by behavioral contrast sensitivity, the physiological ratios were consistently smaller than those measured behaviorally. The authors also found no clear relationship between interocular ratios of contrast sensitivity measured physiologically and those determined psychophysically.

More recently, Shooner et al. (2015) analyzed multiunit recordings in amblyopic macaques, and found that for some macaques, behavioral measures of the contrast sensitivity function (CSF) were much more impaired than the neural measure of V1 sensitivity as a function of SF. In one animal, contrast sensitivity in the AE was so reduced that behavioral thresholds could not be measured above 3 cycles per degree (cpd), yet neural sensitivity still showed strong discriminability for a 4 cpd stimulus. Similar discrepancies between behavioral and physiological measures have been reported in humans with strabismic and anisometropic amblyopia (Barnes et al., 2001).

A growing body of research now shows that perceptual impairments in amblyopia cannot be fully explained by V1 dysfunctions alone. One possibility is that alterations in V1 responses tend to be more modest than their behavioral equivalent because higher visual areas also contribute to behavioral performance. Consistent with this view, receptive field structure and orientation tuning in V2 show abnormalities that are more pronounced than those observed in V1 and correlate strongly with behavioral impairments (Bi et al., 2011; Tao et al., 2014). Amblyopic individuals also exhibit impairments across a wide range of visual functions typically associated with higher-order cortical processing that reflect disruption in global integration and top-down visual mechanisms that are not easily accounted for by early visual processing alone. This has led to the view that amblyopic visual dysfunctions might start in V1 and are subsequently transformed by higher visual areas.

## 4 V1 and retinotopic extrastriate cortex are necessary and sufficient to explain distortions in amblyopia

Although V1 is known as the first disrupted cortical level in amblyopia, converging evidence indicates that functional anomalies extend beyond early-stage processing impairments. Results from Molaei et al. (2025b) provide indirect evidence for the involvement of higher-level integration processes in perceptual distortion in amblyopia. Even though V1 feature maps are known to be spatially interdependent, they found that behavioral distortion maps of different feature dimensions (e.g., position, orientation, spatial frequency) were largely independent from one another.

This observation places an important constraint on a purely V1-based account of distortions in amblyopia. If perceptual distortions were only determined by a common disruption of V1 feature maps, one would expect distortions across different feature dimensions to be correlated across the visual field, thus reflecting a shared underlying encoding error. The absence of such correlations instead suggests that distortions cannot be explained solely by V1 abnormalities. Rather, the absence of correlation suggests that feature-specific representations encoded in V1 are differently transformed by downstream processing stages, resulting in idiosyncratic perceptual distortions for each feature map.

Beyond basic visual function, amblyopes also exhibit profound impairments in higher-order visual tasks that require segregating visual information from background noise (i.e., global motion, figure-ground segmentation, and contour integration; Mansouri and Hess, 2006). Extrastriate visual regions, such as V2, V4, medial temporal cortex (MT+), and lateral occipital complex (LOC), play a crucial role in integrating visual information across time and space. The nature of these impairments suggests a breakdown in the spatial and temporal coordination of local cues to form coherent percepts. We will refer to these impairments as integration-level distortions. That is, the second component of a broader dual-level model of amblyopia, in which visual distortions arise from both altered early-stage local features encoding in V1 and integration-level distortions mediated by altered spatial integration mechanisms within retinotopic extrastriate visual cortex.

We focus specifically on retinotopic visual cortex because amblyopic distortions exhibit a clear retinotopic signature: they are local (i.e., patchy) not global, tied to retinal location (i.e., position specific), vary with orientation, SF and contrast, and are present across various tasks (Bedell and Flom, 1981; Barrett et al., 2003; Sireteanu et al., 2008b; Molaei et al., 2025b). These properties are hallmarks of early and intermediate visual maps (Sereno et al., 1995; Engel et al., 1997; Dumoulin and Wandell, 2008) and are most naturally explained by abnormalities in retinotopically organized cortex.

Although higher-order regions such as inferotemporal or parietal cortex are typically associated with more abstract, and spatially coarse spatial representations (Wandell et al., 2005; Wandell et al., 2007; Silver and Kastner, 2009), it is important to note that top-down processes such as spatial attention can also modulate neural responses in a retinotopically specific manner within early and intermediate visual cortex. Attention is unlikely to be the primary source of amblyopic distortions, as these exhibit stable spatial and feature-specific structure. However, top-down attentional biases may modulate how distorted signals are selected and integrated, thereby influencing the magnitude or perceptual expression of these distortions. Together, these observations suggest retinotopic extrastriate areas (e.g., V2-V4, LOC) as critical substrates for the amplification and spatial structuring of amblyopic distortions.

### 4.1 Contour and global shape integration distortions in human amblyopia

Multiple studies using Gabor-based contour integration tasks found that both strabismic (Hess et al., 1997) and anisometropic (Hess and Demanins, 1998; Norcia et al., 2005) amblyopes are significantly impaired in perceiving global contours and shape. Both the AE and, in some cases, FE show elevated performance threshold compared to control eyes (Kovács et al., 1996; Hess et al., 1997; Kovács et al., 2000; Mussap and Levi, 2000; Chandna et al., 2001; Simmers and Bex, 2004; Levi et al., 2007; Jiang et al., 2023). Impairments in contour detection also seems to depend partly on the spatial scale of the stimulus (i.e., elements size and SF)(Levi et al., 2007; Jiang et al., 2023). Notably, amblyopes present impaired contour detection even when orientation discrimination thresholds for single Gabor patches are normal (Mansouri et al., 2004; Simmers and Bex, 2004), suggesting that the limitation must arise from disrupted global integration rather than degraded local encoding of orientation.

Beyond contours, amblyopes also have issues integrating global shape information, particularly when signal elements are embedded into background noise like in Glass patterns (Rislove et al., 2010). These findings point to a disruption in the ability to extract coherent structures from noise, a process normally associated with areas V2, V4 and LOC (Braddick et al., 2000; Pasupathy and Connor, 2001; Altmann et al., 2003; Ito and Komatsu, 2004; Nandy et al., 2013). Cortese et al. (2009) used fMRI with performance-matched static Glass pattern stimuli and reported attenuated AE-driven activity in LOC, accompanied by abnormal interaction between early visual areas V1 & V2 and extrastriate areas.

These failures of integration are not limited to conditions requiring noise segregation. Amblyopes also show abnormal processing of global shape (Levi et al., 1999) and global orientation (Popple and Levi, 2000; Norcia et al., 2005) even when signal elements are presented without noise. Tasks involving radial frequency patterns, which require the integration of distinct contour segments into smooth closed forms, are impaired in amblyopia (Hess et al., 1999; Dallala et al., 2010). This suggests amblyopia affects the encoding of shapes and contour structure even when noise segregation is not required, indicating fundamental disruptions in global form processing beyond early visual processing.

### 4.2 Global motion integration distortions in human amblyopia

Neuroimaging and psychophysical evidence point to abnormalities extending beyond early visual areas to include motion-sensitive extrastriate cortex. Using visual tracking of moving targets combined with fMRI, Secen et al. (2011) reported reduced activation in MT + for both eyes of amblyopic participants relative to control. Consistent with this, Wang et al. (2017) reported weaker BOLD activation in strabismic amblyopes during a motion salience-driven attention task when stimuli were presented to the AE. Similarly, direction discrimination of high-level random-dot kinematograms (RDKs) has been associated with lower MT + activation in both eyes of children with strabismic and anisometropic amblyopia (Ho and Giaschi, 2009). Simmers et al. (2006) investigated the perception of radial and rotational motion, patterns usually associated with higher visual areas such as MSTd. They reported motion perception impairments in amblyopes that could not be explained by reduced stimulus visibility. Reduced performance on the global motion task persisted even after accounting for low-level visual impairments, indicating that disruptions in global motion processing must arise at stages beyond V1.

To better understand the mechanisms underlying integration-level limitations, Mansouri and Hess (2006) used a global motion direction discrimination task that systematically varied SNR. They showed that AE can integrate motion information normally when the stimulus only contains coherent motion (i.e., signal), but that performance deteriorates rapidly with the introduction of motion noise. Echoing global shape results, this pattern suggests that amblyopic visual systems are less tolerant to noise and that the impairments in global motion performance do not simply reflect degraded early-stage feature encoding, but could instead implicate altered noise segregation mechanisms in extrastriate areas such as MT + .

This distinction between signal integration and noise segregation has important implications. In motion integration tasks that do not require noise segregation, such as plaid motion perception, amblyopic performance is largely preserved. However, in tasks that require differentiating coherent motion from random noise, such as RDKs, performance of amblyopes is worse than controls. This difference likely reflects the engagement of distinct networks supporting these motion tasks. Using fMRI, Thompson et al. (2012) showed that plaid motion perception engages a broader compensatory network of areas possibly involving ventral V3 and the pulvinar, whereas global dot motion, which requires signal-to-noise segregation, relies primarily on area hMT+. This suggests that amblyopia might selectively disrupt motion integration networks related to area MT+, while preserving some ventral and subcortical compensatory mechanisms.

### 4.3 A dual-level model of perceptual distortions in amblyopia

Evidence from human studies confirms that integration deficits in extrastriate areas in amblyopia are not mere extensions of low-level feature encoding impairments. Amblyopic observers can integrate signal information comparably to control individuals in some tasks under noise-free conditions, yet their performance quickly deteriorates once noise is introduced. This pattern points to a specific vulnerability in segregating signal from background noise, a function that depends on integration mechanisms across extrastriate visual cortex. Amblyopic performance might not be constrained by visibility, but rather by impaired coordination of signals across space and time.

**Figure 7:**
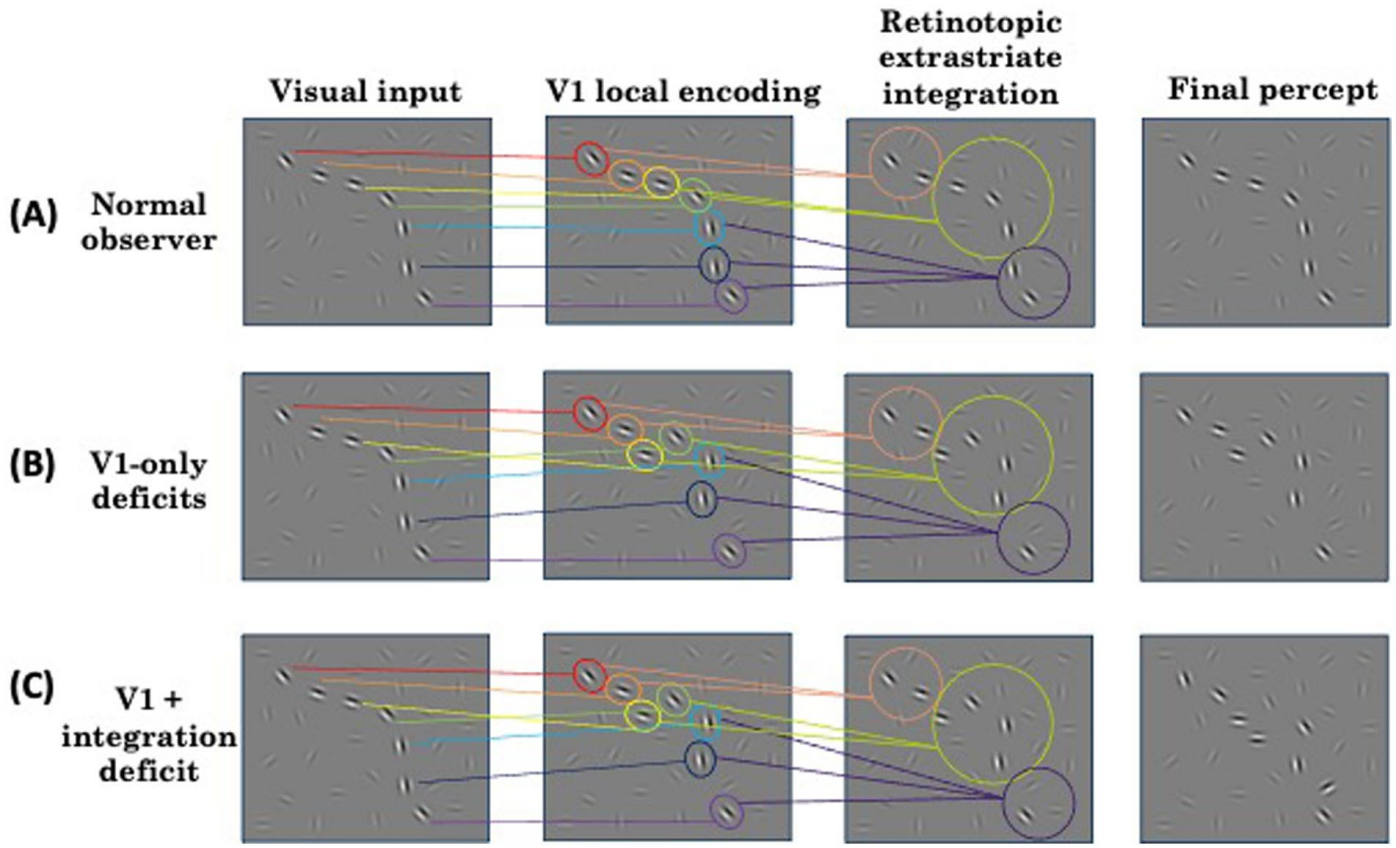
Dual-level model of perceptual distortions in amblyopia. Schematic illustration of how distortions in amblyopia may emerge from the interaction between altered V1 local encoding and impaired spatial integration in retinotopic extrastriate cortex. Each row represents a different hypothetical stage of processing from visual input to final percept. Colored circles illustrate receptive field across cortical space. **(A)** Normal observer. Local visual features are accurately encoded in V1 and reliably integrated across retinotopic extrastriate areas. Despite the presence of noise in the visual input, integration mechanisms correctly segregate signal from background, allowing accurate contour identification. **(B)** V1-only deficits. Local feature encoding is degraded in V1, introducing positional variability and reduced precision of feature representation. However, intact extrastriate integration mechanisms partially compensate for these irregularities, resulting in a final percept that may appear slightly distorted but still recognizable. **(C)** V1 & integration deficits (dual-level model). Local encoding irregularities in V1 interact with impaired spatial integration in extrastriate cortex. Integration mechanisms operate on unreliable or spatially inconsistent inputs, causing noise elements to be incorrectly incorporated into the signal. This amplifies small encoding errors and produces structured perceptual distortions.

Together, the reviewed findings support a dual-level model of amblyopic perceptual distortions (Figure 7). At the first level, abnormalities in early visual cortex degrade the fidelity and spatial precision of local feature representations. Alterations in retinotopy, ocular dominance organization, spatial frequency tuning, and response reliability reduce the accuracy with which local visual features are encoded. These early-stage irregularities introduce variability and positional uncertainty in the neural representation of visual information.

At the second level, these distorted local signals are integrated across space and time within retinotopically organized extrastriate visual areas. Integration processes involved in contour grouping, global form perception and motion coherence rely on coordinated pooling of local feature information across distributed neuronal populations. When local feature representations are degraded or spatially inconsistent, integration mechanisms operate on unreliable inputs, transforming or amplifying small encoding errors intro structured perceptual distortions. These transformations are unlikely to be purely linear but instead might reflect nonlinear pooling or gain-control operations that can progressively amplify modest encoding irregularities across the visual hierarchy.

Several candidate computations may contribute to this transformation. Spatial summation over enlarged or poorly tuned receptive fields could cause extrastriate neurons to pool signals from neighboring locations with mismatched feature preferences (e.g., preferred spatial location), converting local positional uncertainty into contour waviness or fragmentation. Consistent with this hypothesis, imaging studies in humans with amblyopia have reported enlarged receptive fields, disrupted retinotopic organization and increased positional variability across the visual cortex (Clavagnier et al., 2015; Farivar et al., 2019). Clavagnier et al. (2015) further demonstrated that retinotopic abnormalities observed in V2 and V3 could not be fully explained by altered V1 sampling alone, suggesting that higher-order integration processes contribute independently to the transformation of amblyopic representations. In parallel, electrophysiological recordings in amblyopic macaques showed that V2 neurons exhibit abnormal spatial organization of their receptive-field subfields (i.e., integrated V1 inputs), disrupting how neighboring visual features are normally integrated across the visual field (Tao et al., 2014). Specifically, the spatial arrangement of receptive subfields was less homogeneous both within individual V2 neurons and across nearby neuronal population, suggesting that local feature relationships are encoded less consistently in amblyopic extrastriate cortex. These results provide a plausible neural substrate through which modest V1 encoding irregularities can be transformed into larger-scale perceptual distortions.

Altered gain-normalization could further amplify weak or noisy inputs, particularly when signals must be segregated from background noise. Studies have previously shown that this is a condition under which amblyopic performance deteriorates quickly (Kovács et al., 2000; Chandna et al., 2001; Simmers et al., 2003; Kiorpes et al., 2005; Aaen-Stockdale and Hess, 2008; El-Shamayleh et al., 2010). Contextual modulation may also be impacted, reducing the ability of extrastriate neurons to enhance coherent structure while suppressing irrelevant surrounding information. Consistent with this idea, amblyopic observers exhibit decreased performance in contour integration and global processing tasks requiring signal segregation in noisy elements, even when stimuli are made fully visible to the AE by increasing contrast or matching spatial frequency to visibility for example. This means that even when correcting for V1 limitations, integration deficits remain (Mansouri et al., 2004; Constantinescu et al., 2005; Simmers et al., 2005; Jiang et al., 2023). The dual-level model does not require that one of these computations acts in isolation. Rather, it proposes that these operations act on spatially inconsistent feature representations inherited from early retinotopic cortex, such that modest encoding irregularities in V1 are progressively reshaped by higher-level integration.

Within our proposed framework, perceptual distortions emerge not from a single locus of dysfunction, but from the interaction between altered local encoding and integration-level distortions across the visual hierarchy. Since global percepts depend on the coordinated activity of large neuronal populations, even subtle irregularities in early feature maps can propagate through successive stages of processing, producing distortions reported by amblyopes. This framework predicts that distortions should vary across individuals, be spatially heterogeneous, feature specific and stimulus dependent. Distortions should also be most severe in tasks requiring pooling across locations or segregating signal from noise. Conversely, if distortions were fully explained by V1 encoding abnormalities alone, distortion maps for different feature dimensions should show stronger spatial correspondence than currently observed (Molaei et al., 2025b).

The proposed framework also generates several experimentally testable predictions. First, perceptual distortions severity should become more apparent as visual tasks place greater demands on spatial integration across distributed neuronal populations. Distortions should therefore be most evident during contour integration, global form and motion tasks requiring pooling across space or segregation of signal from noise. Second, the magnitude of perceptual distortions should exceed what would be predicted from V1 encoding deficits alone, reflecting downstream amplifications and transformations of altered feature representations within extrastriate cortex. Third, impairments in global integration should partially persists even when differences in stimulus visibility are equated between the AE and FE. That is because higher-level integration mechanisms would continue to operate on spatially inconsistent V1 representations.

Although the present framework emphasizes feedforward transformations from V1 to retinotopic extrastriate cortex, top-down influences such as attention may also modulate the expression of perceptual distortions. A growing literature suggests that amblyopia is associated with atypical spatial attention (Sharma et al., 2000; Popple and Levi, 2008; Verghese et al., 2019; Zhou and Thompson, 2025), which may affect the efficiency with which signals are selected and integrated under noisy conditions. Within the dual-level framework, attentional mechanisms could influence the weighting of spatial signals during pooling in extrastriate cortex, potentially amplifying or mitigating distortions depending on task demands. Attentional modulation does not replace early encoding deficits but may interact with them, shaping how degraded feature representation are integrated across space.

Finally, this framework helps reconcile the apparent mismatch between relatively modest physiological abnormalities observed in V1 and the often more structured distortions reported by amblyopic observers. Rather than reflecting isolated deficits at a single processing stage, amblyopia distortions may reflect cumulative transformations as signals are progressively pooled and integrated across retinotopic cortex.

## 5 Discussion

As we have seen, the literature suggests that perceptual impairments in amblyopia originate from dual levels of cortical dysfunction: unreliable local features encoding within early visual areas and integration-level distortions within higher retinotopic visual regions. This relationship reveals an important methodological caveat that must be considered when interpreting extrastriate abnormalities in amblyopia. Many neurophysiological and neuroimaging studies examining extrastriate cortex in amblyopia have not explicitly controlled for differences in V1 input strength or representational fidelity between the AE and FE, for example by equating stimulus contrast, visibility or response magnitude (Simmers et al., 2003; Simmers et al., 2006). As a result, some abnormalities observed in extrastriate cortex may reflect degraded feedforward input inherited from earlier visual areas rather than intrinsic dysfunction within extrastriate cortex itself.

This limitation does not contradict the proposed framework. The dual-level model does not require extrastriate deficits to arise independently from V1 abnormalities. Rather, it proposes that distorted or spatially inconsistent representations originating in early visual cortex are progressively transformed by integration mechanisms within retinotopically organized extrastriate cortex. In this view, extrastriate impairments may emerge from the combination of inherited feedforward distortions and altered integration-level processing acting on degraded inputs. This methodological limitation may have contributed to the ongoing ambiguity regarding the locus of amblyopic dysfunction. Distinguishing between these possibilities remains an important direction for future work and will require experimental paradigms that explicitly control for V1 response strength and visibility across eyes.

The idea that amblyopia reflects dysfunction beyond a single cortical locus is not new. Previous work has emphasized that amblyopic deficits can arise from distributed alterations across the visual system rather than isolated impairments within V1. For example, psychophysical and neurophysiological evidence reviewed by Levi (2006) suggests that abnormalities emerging in early visual cortex can have consequences that propagate through subsequent stages of processing. Similarly, Hess and Thompson (2015) propose a two-stage model of binocular contrast gain control in which interocular suppression and imbalances in excitatory and inhibition circuits determine how signals from the two eyes are combined.

However, these accounts differ with the proposed framework here in the aspects of visual processing that they seek to explain and their level of integration. Levi (2006) emphasizes that amblyopic deficits originate in early visual cortex but are subsequently amplified through downstream processing stages. The review also provides evidence for abnormalities in higher-level functions such as global form, motion integration and crowding. While this work clearly supports a hierarchical view of amblyopic deficits, it remains largely descriptive, focusing on how impairments are present across different perceptual tasks rather than providing a unified account of how these perceptual deficits arise from transformations across the visual hierarchy. In contrast, models such as Hess and Thompson (2015) are primarily concerned binocular combination, emphasizing interocular suppression and contrast gain control as determinants of visual performance in amblyopia. Neither framework addresses how spatial information is not properly encoded in amblyopia.

More generally, existing theories of amblyopia tend to focus on specific stages of visual processing, including early encoding abnormalities, population response dynamics or higher-level perceptual impairments. In this sense, classical encoding, compensatory pooling and Bayesian decoding frameworks may each capture different components of amblyopic dysfunction rather than representing mutually exclusive explanations. However, these theories typically emphasize isolated stages of processing and do not mutually link these levels into a single mechanistic framework. As a result, they cannot fully explain how encoding irregularities within retinotopically organized V1 maps (e.g., spatial frequency) are propagated, integrated and transformed into the perceptual distortions reported by amblyopes.

The framework proposed here addresses this gap by providing a unified explanation of how distortions emerge from the interaction between local encoding irregularities and higher-order integration mechanisms. Rather than focusing on a single processing stage, it seeks to explain how spatially structured distortions arise from the transformation of altered feature representations across the cortical hierarchy. In this view, amblyopic perceptual distortions are not solely a consequence of reduced signal strength or binocular imbalance but instead emerge from the integration and transformation of spatially altered representations within extrastriate cortex. In other words, early encoding abnormalities provide the distorted inputs, while extrastriate integration mechanisms amplify, reshape, and contextualize these signals. These transformations likely involve nonlinear pooling, contextual modulation and gain-control operations acting on spatially inconsistent representations inherited from early visual cortex. This framework also provides a natural explanation for the heterogeneity of amblyopic perceptual distortions (Molaei et al., 2025a; Molaei et al., 2025b). Rather than reflecting a single underlying deficit, different distortion patterns may arise from distinct combinations of encoding irregularities and integration-level transformations across the visual hierarchy.

A comprehensive understanding of amblyopic vision must therefore consider both stages of disruption within the visual hierarchy. One plausible unifying factor could be the failure of binocular fusion. Binocular integration impairments could compromise interocular matching in early cortical processing but also degrade the brain’s ability to integrate monocular signals into coherent global percepts. When the two eyes encode spatial information differently, stable binocular correspondence becomes difficult to establish, potentially leading to rivalry and interocular suppression during binocular viewing.

Although interocular suppression is a central feature of amblyopia, its relationship to perceptual distortions remains unclear. Most distortion paradigms assess perception monocularly through the AE, meaning that distortions are perceived even in the absence of direct binocular competition from the FE. This suggests that perceptual distortions cannot be explained solely by active interocular suppression. However, distorted monocular representations may themselves contribute to failures of binocular fusion. If the two eyes encode information differently, binocular correspondence becomes difficult to establish, potentially leading to rivalry or suppression under binocular viewing conditions.

One major component of the functional alterations in amblyopia is abnormal binocular interaction. Over the years, binocular measures have been shown to be good indicators to distinguish both the severity and subtypes of amblyopia (McKee et al., 2003; Levi, 2006; Piano et al., 2015). Binocular fusion failures in amblyopia arise from the brain’s inability to combine mismatched inputs from the AE and FE. As a result, amblyopes often lack normal stereopsis (for review, see (Levi et al., 2015)) and exhibit suppression of AE input under binocular viewing conditions (Travers, 1938; Smith et al., 1985; Li J. et al., 2011; Hess et al., 2014).

Neurophysiological and imaging studies consistently show that binocular neurons are less responsive and numerous in amblyopic visual cortex (Hubel and Wiesel, 1965; Kiorpes et al., 1998; Lee et al., 2001; Conner et al., 2007). Dichoptic stimulation also reveals binocular suppression of V1 neurons in both animal models and human amblyopes (Sengpiel et al., 1995; Sengpiel and Blakemore, 1996; Smith et al., 1997; Zhang et al., 2005; Farivar et al., 2011), hinting at a functional disconnect in the integration of AE and FE inputs.

We propose that this breakdown in binocular fusion may represent one mechanism linking early-stage local feature distortions with later-stage integration-level failures in amblyopia. Binocular fusion depends on the brain’s ability to match information from both eyes. In amblyopia, distorted representation of V1 feature maps, such as shifted retinotopy or reduced SF sensitivity, leads to mismatched monocular representations. These mismatches disrupt interocular correspondence, preventing proper fusion. This results in suppression of the amblyopic signal and impaired integration across space and time.

From this perspective, integration-level distortions in amblyopia—whether in contour, form or motion perception—may come from a more fundamental failure in binocular matching that is driven, at least partially, by monocular distortions. We hypothesize these integration-level impairments originate from differential organizations in V1 representation and subsequently propagate to higher-level retinotopic areas responsible for combining local features into coherent percepts. Since global percepts rely on the pooling of local signals across many neurons, even subtle mismatches at early stages can be amplified downstream, leading to perceptual distortions.

In our view, a hallmark of amblyopia impairment—reduced or absent binocular integration—may arise from perceptual distortions that are monocular in origin, emerge within V1, and propagate through retinotopically organized extrastriate cortex. From this perspective, binocular vision failure is not merely a downstream consequence of amblyopia, but a mechanism through which early-stage representational distortions give rise to higher-level perceptual impairments.

Consistent with this, several studies have reported strong associations between the severity of perceptual distortions and binocular function failures, including impaired fusion and reduced stereoacuity (Kwon et al., 2014b; Hussain et al., 2015; Piano et al., 2015, 2016). These findings suggest that mismatches introduced by distorted monocular representations may compromise binocular correspondence.

Our perspective leads to a new testable hypothesis: if the distorted local representations within the AE were corrected, then binocular correspondence would be restored. A substantial body of work demonstrates that residual cortical plasticity persists in late childhood and adulthood, as repeated practice on visual tasks using the AE can improve performance in amblyopic adult observers (e.g., Vernier acuity, positional acuity, contrast-defined and luminance-defined letter identification; Wick et al., 1992; Epelbaum et al., 1993; Levi and Polat, 1996; El Mallah et al., 2000; Li and Levi, 2004; Chung et al., 2008). These findings have motivated the development of novel therapeutic approaches that move beyond monocular training and instead target binocular interaction and cooperation. In particular, dichoptic and perceptual learning paradigms aim to rebalance interocular inputs by presenting complementary stimuli to each eye, such that successful task performance requires binocular integration (Levi and Li, 2009; Hess et al., 2010a; Li R. W. et al., 2011; Fu et al., 2022). While these approaches have shown promise in improving binocular function, they are primarily designed to reduce interocular suppression and enhance binocular cooperation, rather than explicitly targeting spatial distortions. However, we predict that by extension, training protocols specifically designed to target spatial distortions would lead to measurable gains in binocular function. It is anticipated that, following either transient correction or extensive retraining, binocular integration would may be enhanced but would likely need additional corrected experience and/or targeted training to fully recover.

Novel perceptual learning protocols could be designed around individualized distortion correction and progressive retraining. First, spatial distortion patterns would be mapped across the visual field using tasks probing position, orientation and spatial frequency tuning similar to those proposed by Molaei et al. (2025b). These measurements could then inform the design of personalized dichoptic tasks in which successful performance requires combining complementary signals from the two eyes at locations or feature ranges that are known to be distorted. For example, contour fragments, oriented elements or SF-defined stimuli could be distributed across the two eyes such that task completion is only possible if accurate binocular correspondence is re-established. By systematically reinforcing correct alignment between monocular inputs, this approach aims to recalibrate the integration of spatially altered representations across the visual hierarchy. More broadly, it provides a concrete pathway for translating distortion mapping into targeted, mechanism-based rehabilitation strategies.

This approach also provides an experimental test of the proposed framework. If spatial distortions in the AE contribute to impaired binocular correspondence, then individualized correction of these distortions should improve binocular integration selectively at the corrected visual-field locations or feature dimensions. The dual-level model would predict that binocular performance improves most strongly where AE distortions were corrected, with the magnitude of improvement scaling with the initial distortion. Conversely, if AE distortions are corrected for but binocular correspondence remains unchanged, this would suggest that spatially distorted AE representations are not a causal bottleneck for binocular combination. It would also weaken the proposed link between local encoding irregularities and higher-level integration failure.

A key factor underlying the persistence of these distortions is the nature of cortical plasticity in the visual system. Amblyopia develops during sensitive periods of development, when visual experience shapes the formation and refinement of cortical circuits. During these periods, abnormal binocular inputs, whether through monocular deprivation, significant refractive asymmetry, or severe strabismus, can lead to long-lasting structural and functional changes in cortical architecture. Although some degree of plasticity persists in adulthood, it differs mechanistically from developmental plasticity and is generally more limited in its capacity to reorganize large-scale cortical representations (Berardi et al., 2000; Hooks and Chen, 2007; Bavelier et al., 2010; Hensch and Quinlan, 2018). This constraint can help explain why conventional treatments such as occlusion therapy can improve visual acuity, by strengthening monocular input from the AE, yet often fail to fully restore binocular function or eliminate perceptual distortions, which depend on the coordinated development and calibration of binocular and integration mechanisms during earlier critical periods (Constantinescu et al., 2005; Hayward et al., 2011; Giaschi et al., 2013; Giaschi et al., 2015; Piano et al., 2015; Jia et al., 2022).

In this context, perceptual learning combined with non-invasive electromagnetic stimulation approaches (e.g., repetitive transcranial magnetic stimulation, anodal transcranial direct-current stimulation, transcranial random-noise stimulation) have been proposed as means to enhance residual plasticity in the adult visual system (Hess and Thompson, 2013, 2015; Householder et al., 2025). By modulating cortical excitability and promoting experience-dependent reorganization (Birch and Duffy, 2024), these approaches may help re-engage integration mechanisms within extrastriate cortex, providing a potential method to reduce spatial distortions and improve binocular correspondence.

## 6 Conclusion

Taken together, the evidence reviewed here indicates that amblyopia is not characterized by a gross loss of early visual cortical function, but by systematic impairments in the balance, fidelity and spatial organization of visual representations. Alterations in the LGN and V1 are reliable and measurable, yet they are typically modest in magnitude and insufficient, on their own, to account for the severity and structure of perceptual distortions observed in amblyopia. Responses driven by the AE are often noisier, less precise and more spatially irregular than those driven by the FE, producing interocular imbalances rather than uniform loss.

We propose a dual-level model of amblyopia, in which perceptual distortions arise from the interaction between (1) early-stage impairments of local visual features within V1 and (2) integration-level distortions of visual features in downstream, retinotopically organized extrastriate cortex. Within this framework, impairments in early retinotopic representations are expected to constrain downstream extrastriate processing. Rather than being contained within V1, early impairments are amplified as signals are pooled across space and time, giving rise to pronounced impairments in global form, motion, and structure perception. In this view, amblyopic perceptual deficits emerge from failures of integration building on irregular early representations, rather than from isolated dysfunction at a single cortical stage.

Understanding amblyopia therefore requires a multilevel framework in which early-stage representational impairments and later-stage integration failures are treated as interacting components of a single system. From this perspective, restoring visual function in amblyopia will likely require not only improving early feature encoding, but also recalibrating the integrative computations that transform local signals into coherent global percepts.

The multilevel framework proposed here might not be unique to amblyopia. It may also generalize to other conditions characterized by reduced sensitivity to complex visual information (e.g., contour or motion processing deficits). However, amblyopia provides a uniquely informative model system since abnormal visual experiences during development produce systematic, retinotopically organized distortions while preserving the organization of the visual cortical hierarchy. This combination allows the relationship between local encoding deficits and global perceptual distortions to be examined within the same visual system, something that might be difficult to achieve in conditions involving widespread degeneration or non-specific sensory losses.
